# The value of adjuvant chemotherapy in stage II/III colorectal signet ring cell carcinoma

**DOI:** 10.1038/s41598-020-70985-0

**Published:** 2020-08-24

**Authors:** Zhuang Zhao, Na Yan, Shu Pan, Dun-wei Wang, Zhi-wen Li

**Affiliations:** Department of Anesthesiology, First Hospital of Jilin University, Changchun, 130021 Jilin China

**Keywords:** Cancer, Oncology, Risk factors

## Abstract

This study aimed to assess the benefit of postoperative adjuvant chemotherapy in stage II–III colorectal signet ring cell carcinoma (SRCC). Qualified postoperative patients were extracted from Surveillance, Epidemiology, and End Results (SEER) database from 2004 until 2015. We collected 1675 patients in the research, and 936 patients were subjected to adjuvant chemotherapy group. The proportions of married status, male, rectal cancer, grade III/IV, AJCC stage III and radiotherapy were higher; While, the rates of white race, ≥ 65 years old and located in cecum–transverse colon were lower in patients of chemotherapy group compared to no chemotherapy group (all *P* < 0.05). K-M plots revealed significantly better OS of adjuvant chemotherapy group than no chemotherapy group (*P* < 0.001). Meanwhile, there was no significantly different in CSS between the two groups (*P* = 0.93). However, after adjusting for confounding factors by multivariable Cox regression analysis, receipt of postoperative chemotherapy was still associated with better CSS and OS (CSS: hazard ratio [HR] = 0.719, 95% CI 0.612–0.844, *P* < 0.001) ; (OS: HR = 0.618, 95% CI 0.537–0.713, *P* < 0.001). Patients with stage II/III colorectal SRCC could receive survival benefit from postoperative adjuvant chemotherapy.

## Introduction

Colorectal cancer (CRC) ranks the third of cancer-associated death, causing great health burden globally^1^. The diverse pathological types of CRC have been uncovered to be having correlation with various clinical parameters and patient survival, with adenocarcinoma being most prevalent type^2,3^. Signet-ring cell carcinoma (SRCC) is a relatively rare histological subtype, consisting of 0.1–2.6% of CRC patients^4,5^, defined as the abundant presence of intracellular mucin in over 50% cells according to WHO^6,7^.

SRCC is considered as a distinct pathological subtype in CRCs. A series of differences among colorectal SRCC, mucinous adenocarcinoma (MC) and non-mucinous adenocarcinoma (NMC) have been consistently reported. To be specific, SRCC has been reported to be associated with younger age at diagnosis, more advanced stage and poorer clinical outcomes than MC and NMC^7–9^. In addition, massive lymphatic involvement, higher frequency of multiple metastatic organs and greater risks of peritoneal metastases are more commonly seen in SRCC^9^.

Because SRCC is relatively rare, there is a lack of consensus on therapeutic guidelines due to the difficulty in conducting large randomized controlled trials^5^. At present, surgical intervention is still the optimal option for colorectal SRCC patients. Moreover, the combined application of other therapeutics has been increasing, especially chemotherapy^10^. Hugen et al. have assessed the efficacy of adjuvant chemotherapy in colorectal SRCC, who further indicated the benefit of adjuvant chemotherapy in stage III SRCC patients^11^. Meanwhile, by analyzing the distinct metastatic patterns of colorectal SRCC toward different sites, Tao et al. have demonstrated better survival of received chemotherapy in metastatic colorectal SRCC patients^12^. However, there were still some studies showed that colorectal SRCC responded poorly to chemotherapy^13,14^. Thus, clear elucidation of the efficacy of postoperative chemotherapy in colorectal SRCC patients is of great significance.

The SEER database, the most authoritative and largest cancer dataset in North America^15^, records tumor data by covering almost 30% of population in the USA from diverse geographic regions, which could readily represent the population diversity^16^. Therefore, SEER is widely acknowledged as a valuable database for investigation into rare tumors^17–20^. Herein, in the present study, we collected eligible non-metastatic colorectal SRCC patients from SEER database to investigate the influence of adjuvant chemotherapy.

## Materials and methods

### Study population

SEER*Stat v8.3.6 tool (released on August 8th, 2019) was adopted for selecting qualified subjects. Colorectal SRCC patients who were diagnosed from January 1, 2004 to December 31, 2015 were selected from the Incidence-SEER 18 Registries Custom Data (with additional treatment fields). Eligible patients were collected accordingly: (1) primary colorectal SRCC patients; (2) the diagnosis of SRCC was based on (ICD-O-3; coded as 8490/3). Patients were eliminated if they had: (1) more than one primary malignancies; (2) reported diagnosis source from autopsy or death certificate or no pathological diagnosis; (3) no AJCC stage; (4) no surgery; (5) AJCC stage I/IV; (6) no prognostic information. The remaining qualified populations were included, followed by assignment of patients into adjuvant chemotherapy group and no chemotherapy group according to whether they had chemotherapy or not.

### Covariates and endpoint

The following clinicopathological parameters were analyzed: year of diagnosis (2004–2007, 2008–2011, 2012–2015); insured status (uninsured/unknown, any medicaid/insured); age (< 65, ≥ 65); marital status (unmarried, married); gender (female, male); race (black, white or others); primary site(cecum–transverse colon, descending colon–sigmoid, multiple, rectum and unknown); grade (grade I/II, grade III/IV, unknown); tumor size (≤ 5 cm, > 5 cm, unknown); AJCC stage ( stage II, stage III); lymph node dissection (none or biopsy, 1 to 3 regional lymph nodes removed, ≥ 4 regional lymph nodes removed, unknown); chemotherapy (no/unknown, yes) and radiotherapy (no/unknown, yes). The widowed or single (never married or having a domestic partner) or divorced or separated patients were classified as unmarried. The primary tumor site was classified as cecum–transverse colon (including the cecum, appendix, ascending colon, hepatic flexure and the transverse colon), descending colon–sigmoid (including the splenic flexure and descending and sigmoid colons), multiple, rectum and unknown. In addition, the staging of cancer is based on the 6th edition of AJCC stage system, which adapted to patients in the SEER database with a diagnosis time of 2004–2015.

The endpoint of this study was cancer‐specific survival (CSS) and overall survival (OS). CSS was defined as the period from diagnosis to death attributed to colorectal SRCC. OS was defined as the period from diagnosis to death from any cause.

### Statistical analysis

Categorical data were compared by Chi‐square test between chemotherapy and no chemotherapy groups. Kaplan–Meier (K-M) method was adopted for univariate analysis to evaluate whether CSS and OS were different between two groups (log-rank test). Variables with *P* values lower than 0.1 in univariate analysis were incorporated into the multivariate Cox proportional hazard model. SPSS software (version 19.0) (SPSS Inc., Chicago, USA) was employed for statistical analysis, and Graph Pad Prism 5 was utilized for generating survival curve. A two-sided *P* < 0.05 indicated statistical significance.

### Ethics statement

In order to obtain relevant data from the database, we signed the SEER Research Data Agreement (No.19817-Nov2018) and further searched for data according to the approved guidelines. The extracted data were publicly accessible and de-identified, and the data analysis was considered as non-human subjects by Office for Human Research Protection, therefore, no approval was demanded from institutional review board.

## Results

### Patient characteristics

**Figure 1:**
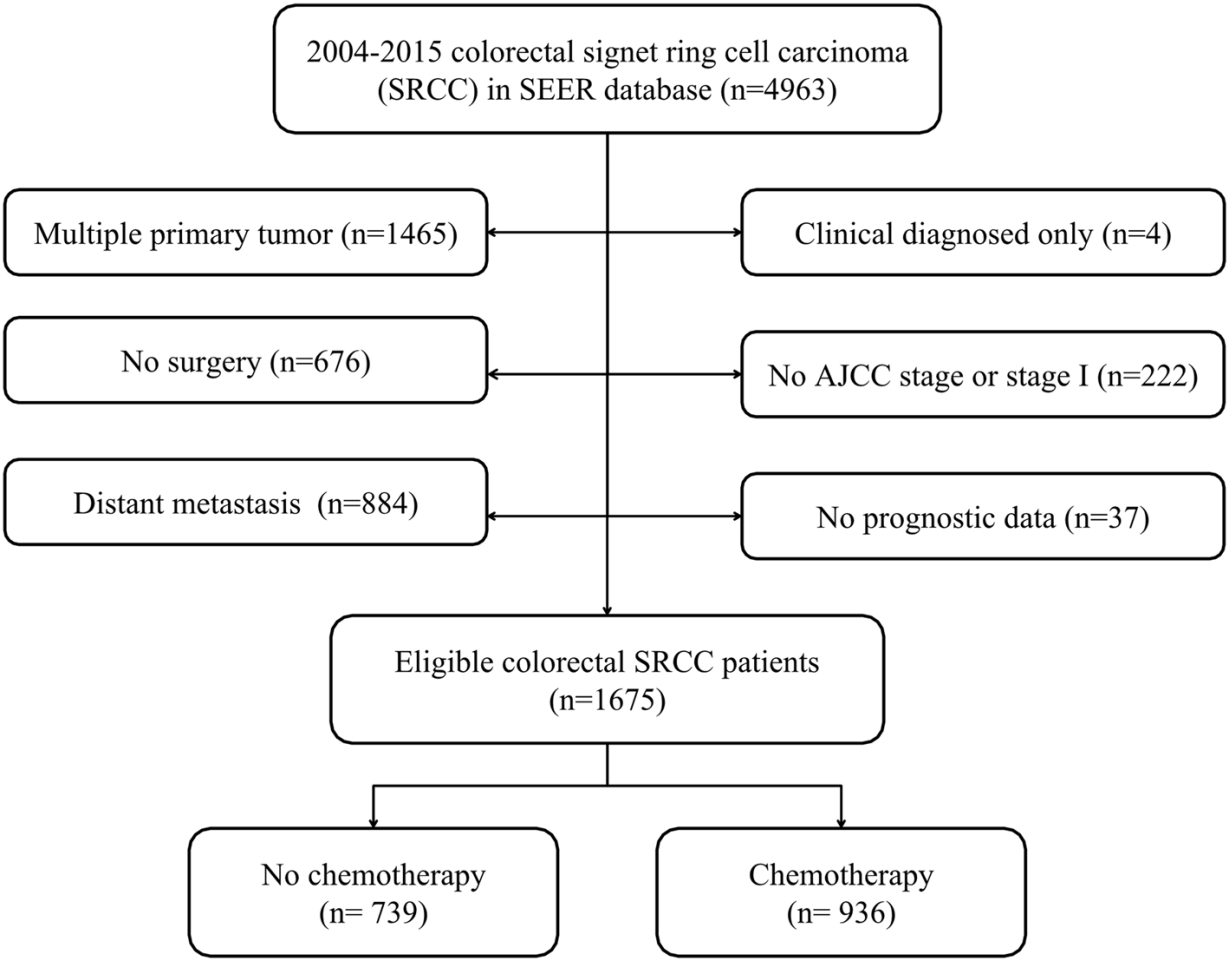
Flow chart of patient selection.

In total, 1675 eligible patients were included in this research, and 936 patients received adjuvant chemotherapy while 739 patients did not. The process of patient selection was displayed in Fig. 1. The demographics, tumor characteristics and therapeutic features of both groups were summarized in Table 1. Except for year at diagnosis, insured status and lymph node dissection, multiple variables were significantly different between the two groups (all *P* < 0.05). There were more patients with married (57.59% vs. 48.44%), male (54.81% vs. 47.36%), located in rectum (29.38% vs. 9.47%), grade III/IV (84.08% vs. 82.41%), AJCC stage III (83.01% vs. 59.27%); less often white race (78.31% vs. 85.66%), ≥ 65 years old (36.65% vs. 73.88%) and located in cecum–transverse colon (52.56% vs. 76.45%) in chemotherapy group compared with no chemotherapy group. Furthermore, more subjects received radiotherapy in adjuvant chemotherapy group (28.31% vs. 2.71%).

**Table 1:** Baseline characteristics of colorectal signet ring cell carcinoma patients included in this study.

Characteristics	No chemotherapy	Chemotherapy	*P*-value
**Year at diagnosis**			0.409
2004–2007	263 (35.59%)	305 (32.59%)	
2008–2011	237 (32.07%)	321 (34.29%)	
2012–2015	239 (32.34%)	310 (33.12%)	
**Insured status**			0.245
Uninsured/unknown	239 (32.34%)	278 (29.70%)	
Any medicaid/insured	500 (67.66%)	658 (70.30%)	
**Age**			< 0.001
< 65	193 (26.12%)	593 (63.35%)	
≥ 65	546 (73.88%)	343 (36.65%)	
**Marital status**			< 0.001
Unmarried	381 (51.56%)	397 (42.41%)	
Married	358 (48.44%)	539 (57.59%)	
**Gender**			0.002
Female	389 (52.64%)	423 (45.19%)	
Male	350 (47.36%)	513 (54.81%)	
**Race**			< 0.001
Black	59 (7.98%)	90 (9.62%)	
White	633 (85.66%)	733 (78.31%)	
Others	47 (6.36%)	113 (12.07%)	
**Primary site**			< 0.001
Cecum–transverse colon	565 (76.45%)	492 (52.56%)	
Descending colon–sigmoid	82 (11.10%)	153 (16.35%)	
Multiple	12 (1.62%)	9 (0.96%)	
Rectum	70 (9.47%)	275 (29.38%)	
Unknown	10 (1.35%)	7 (0.75%)	
**Grade**			0.001
Grade I/II	66 (8.93%)	45 (4.81%)	
Grade III/IV	609 (82.41%)	787 (84.08%)	
Unknown	64 (8.66%)	104 (11.11%)	
**Tumor size**			0.015
≤ 5 cm	317 (42.90%)	418 (44.66%)	
> 5 cm	360 (48.71%)	405 (43.27%)	
Unknown	62 (8.39%)	113 (12.07%)	
**AJCC stage**			< 0.001
II	301 (40.73%)	159 (16.99%)	
III	438 (59.27%)	777 (83.01%)	
**Lymph node dissection**			0.157
None or biopsy	80 (10.83%)	77 (8.23%)	
1–3	18 (2.44%)	19 (2.03%)	
≥ 4	641 (86.74%)	840 (89.74%)	
**Radiotherapy**			< 0.001
No/unknown	719 (97.29%)	671 (71.69%)	
Yes	20 (2.71%)	265 (28.31%)	

### Survival analysis of all patients

**Figure 2:**
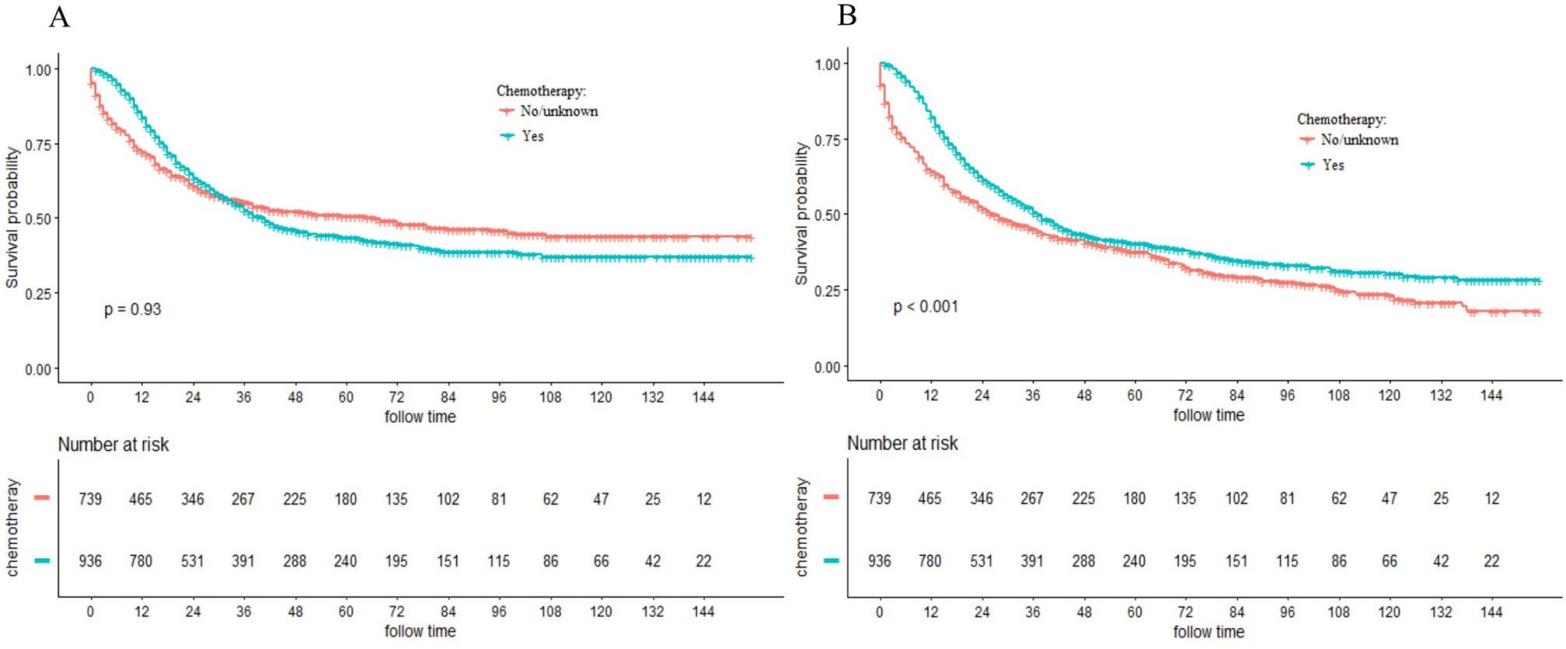
Kaplan–Meier (K-M) curves for cancer-specific survival (CSS) (**A**) and overall survival (OS) (**B**) between adjuvant chemotherapy and no chemotherapy groups.

The median survival time of all included patients was 25.0 months (0–155 months). The 3-, 5- and 10-year CSS rate was 53.47%, 46.14% and 39.53%, respectively. In addition, the 3-, 5- and 10-year OS rate was 47.93%, 39.06% and 26.75%, respectively. K-M plots revealed significantly better OS of adjuvant chemotherapy group than no chemotherapy group (*P* < 0.001). Meanwhile, there was no significantly different in CSS between the two groups (*P* = 0.93).The survival curves of CSS as well as OS were displayed in Fig. 2.

In univariate analysis of CSS and OS, age, marital status, race, primary site, grade, tumor size, AJCC stage, lymph node dissection, chemotherapy and radiotherapy were risk factors for survival (*P* < 0.05), which were later incorporated into the multivariate Cox analysis. As a results, adjuvant chemotherapy was a significantly protective factor for survival (CSS: hazard ratio [HR] = 0.719, 95% CI 0.612–0.844, *P* < 0.001); (OS: HR = 0.618, 95% CI 0.537–0.713, *P* < 0.001). The concrete results of univariate analysis and multivariate analysis were listed in Tables 2 and 3 respectively.

**Table 2:** Univariate analyses of cancer special survival (CSS) and overall survival (OS) for patients.

Variables	CSS		OS	
	χ^2^	*P*-value	χ^2^	*P*-value
**Year at diagnosis**	1.792	0.408	0.425	0.809
2004–2007				
2008–2011				
2012–2015				
**Insured status**	0.100	0.752	0.042	0.838
Uninsured/unknown				
Medicaid/insured				
**Age**	3.228	0.072	48.231	< 0.001
< 65				
≥ 65				
**Marital status**	3.833	0.050	11.387	0.001
Unmarried				
Married				
**Gender**	1.064	0.302	0.253	0.615
Female				
Male				
**Race**	6.898	0.032	4.279	0.118
Black				
White				
Other				
**Primary site**	28.228	< 0.001	17.909	< 0.001
Cecum–transverse colon				
Descending colon–sigmoid				
Multiple				
Rectum				
Unknown				
**Grade**	12.475	0.002	10.475	0.005
Grade I/II				
Grade III/IV				
Unknown				
**Tumor size**	17.228	< 0.001	10.149	0.006
≤ 5 cm				
> 5 cm				
Unknown				
**AJCC stage**	117.678	< 0.001	73.305	< 0.001
II				
III				
**Lymph node dissection**	45.345	< 0.001	49.265	< 0.001
None or biopsy				
1–3				
≥ 4				
**Radiotherapy**	14.163	< 0.001	3.347	0.067
No/unknown				
Yes				
**Chemotherapy**	0.008	0.930	22.324	< 0.001
No/unknown				
Yes				

CSS: cancer‐specific survival; OS: overall survival.

**Table 3:** Multivariate analyses of cancer special survival (CSS) and overall survival (OS) for included patients.

Variables	CSS		OS	
	HR (95% CI)	*P*-value	HR (95% CI)	*P*-value
**Age**		< 0.001		< 0.001
< 65	Reference		Reference	
≥ 65	1.354 (1.163, 1.576)		1.695 (1.478, 1.943)	
**Marital status**		0.378		0.158
Unmarried	Reference		Reference	
Married	0.939 (0.815, 1.080)		0.914 (0.807, 1.036)	
**Race**		0.021		0.022
Black	Reference		Reference	
White	0.901 (0.707, 1.148)	0.399	0.859 (0.691, 1.068)	0.171
Other	1.233 (0.902, 1.686)	0.190	1.126 (0.846, 1.500)	0.415
**Primary site**		0.111		0.032
Cecum–transverse colon	Reference		Reference	
Descending colon–sigmoid	1.039 (0.842, 1.283)	0.718	1.065 (0.883, 1.284)	0.513
Multiple	1.414 (0.827, 2.416)	0.205	1.323 (0.790, 2.215)	0.288
Rectum	1.097 (0.825, 1.458)	0.523	1.182 (0.915, 1.525)	0.201
Unknown	2.106 (1.153, 3.845)	0.015	2.143 (1.278, 3.594)	0.004
**Grade**		0.008		0.008
Grade I/II	Reference		Reference	
Grade III/IV	1.570 (1.136, 2.169)	0.006	1.514 (1.149, 1.997)	0.003
Unknown	1.808 (1.244, 2.628)	0.002	1.638 (1.179, 2.274)	0.003
**Tumor size**		< 0.001		< 0.001
≤ 5 cm	Reference		Reference	
> 5 cm	1.211 (1.043, 1.406)		1.138 (0.998, 1.298)	0.054
Unknown	1.691 (1.338, 2.138)		1.524 (1.230, 1.889)	< 0.001
**AJCC stage**		< 0.001		< 0.001
II	Reference		Reference	
III	3.651 (2.967, 4.491)		2.688 (2.280, 3.171)	
**Lymph node dissection**		< 0.001		< 0.001
None or biopsy	Reference		Reference	
1–3	0.498 (0.312, 0.796)		0.580 (0.382, 0.881)	0.011
≥ 4	0.413 (0.328, 0.520)		0.449 (0.364, 0.553)	< 0.001
**Radiotherapy**		< 0.001		0.447
No/unknown	Reference		Reference	
Yes	1.168 (0.871, 1.567)		1.110 (0.848, 1.454)	
**Chemotherapy**		< 0.001		< 0.001
No/unknown	Reference		Reference	
Yes	0.719 (0.612, 0.844)		0.618 (0.537, 0.713)	

CSS: cancer‐specific survival; OS: overall survival; HR: hazard ratio.

## Discussion

The multidisciplinary management of colorectal SRCC is required to select the optimal therapeutic strategies based on both natural history of tumor and tumor-associated prognostic factors. According to the present international guidelines, no specific therapy is recommended for SRCC histology in clinical practice^21,22^. Surgical intervention is vitally involved in treating localized tumors^5^. However, studies have shown the lower rate of curative resection as well as poorer outcome of colorectal SRCC^14^. Therefore, the application of other therapeutic approaches has been increasing, including chemotherapy^10^. However, relevant researches have shown that colorectal SRCC is relatively insensitive to the commonly applied chemotherapeutics, such as irinotecan, oxaliplatin as well as 5-fluorouracil^13,14,23^. Cabibi et al. have demonstrated that such drug resistance may be due to a low proliferative activity of tumor cells, as the analysis of 15 SRCC samples showed very low levels of Ki-67 expression (a proliferation marker) and weak positivity for thymidylate synthase (key enzyme for DNA synthesis pathways targeted by 5-FU)^13,23,24^.

Conversely, in several large sample-based retrospective studies, we have found that chemotherapy provides significant survival benefits for certain colorectal SRCC populations. Tao Shi et al. found that chemotherapy was related to better survival in metastatic colorectal SRCC^12^. Additionally, the clinical significance of chemotherapy on colorectal SRCC was evaluated in a population-based study involving 1972 patients from 1989 to 2010. The study found that patients with stage III colon SRCC receiving adjuvant chemotherapy had better survival compared to those without chemotherapy (5-year survival rate: 52% vs. 30%)^11^. In the present study, we analyzed 1675 stage II/III colorectal SRCC patients and found that chemotherapy could significantly prolong survival in CSS and OS. This is similar to the previous study. Unfortunately, we were not able to analyze the benefit of adjuvant chemotherapy in high-risk stage II patients specially, due to the low number of patients who received chemotherapy in this group and because the motivation for administration of chemotherapy was not registered.

As a large population based dataset, SEER could be used for cross-sectional assessment in a large number of tumor patients and simultaneously provide long-term follow-up data without inherent institutional bias. Nevertheless, several limitations are unavoidable in our research. First of all, as a retrospective study, the intrinsic selection bias exists in this study^18,20^. Furthermore, the effects of other adjuvant therapy are not assessed, and the specific type of chemotherapeutic regimen is unclear (single agent or doublet). Thus, we are unable to precisely elucidate whether differences exist in terms of adjuvant therapy throughout the study. Thirdly, therapeutic responses as well as recurrence rates are inaccessible from SEER database. Finally, several important prognostic information are unavailable from SEER database, such as: specific number of lymph node dissection ,extramural vascular invasion or obstruction/occlusion status, and Microsatellite stability/Microsatellite instability (MSS/MSI) status .Although it is better to obtain more details, we believed that the present available data from SEER database could fit our research objectives very well. The above concerns should be investigated in future studies.

## Conclusion

In conclusion, our results have shown that stage II/III colorectal SRCC can gain survival benefit from postoperative adjuvant chemotherapy. This is a large population-based study to discuss adjuvant chemotherapy for patients with localized colorectal SRCC, and our present findings might be of significance for disease management and future prospective researches.
